# Binding of Epimedium Flavonoids to Human Serum Albumin: Insights from Spectroscopic and Molecular Docking Studies

**DOI:** 10.5812/ijpr-172525

**Published:** 2026-08-11

**Authors:** Na Wang, Yuwei Wang, Yang Yang, Lele Dong, Yi Zhao

**Affiliations:** 1 Department of Pharmacy, The Second Affiliated Hospital of Xi'an Jiaotong University, Xi'an, 710004, China

**Keywords:** Human Serum Albumin, Epimedin, Flavonoids, Molecular Docking, Multi-spectroscopic Analysis

## Abstract

**Background:**

Epimedin A (EA), epimedin B (EB), and epimedin C (EC), major bioactive flavonoids in Epimedium Herba, have diverse pharmacological activities; however, their interactions with human serum albumin (HSA) remain unclear.

**Objectives:**

This study aimed to investigate the binding mechanisms of EA, EB, and EC to HSA.

**Methods:**

Multispectroscopic techniques, including UV–vis, fluorescence, and synchronous fluorescence spectroscopy, as well as molecular docking and dynamics simulations, were employed under simulated physiological conditions. *Fluorescence spectra* were recorded at 298, 303, and 310 K, and molecular docking was performed with HSA (PDB ID: 1BKE).

**Results:**

All three flavonoids quenched the intrinsic fluorescence of HSA via static quenching, forming 1:1 complexes with binding constants greater than 10^4^ L·mol^-1^ (EB > EA > EC at 298 K). Thermodynamic analysis indicated spontaneous binding driven by hydrogen bonds and van der Waals forces. Synchronous fluorescence showed no significant microenvironmental changes around Trp and Tyr residues. Molecular docking and dynamics simulations suggested ligand-dependent differences in the preferred binding regions of HSA.

**Conclusions:**

These findings provide preliminary in vitro evidence for the binding of epimedin flavonoids to HSA and may inform future pharmacokinetic investigations.

## 1. Background

**Figure 1.**
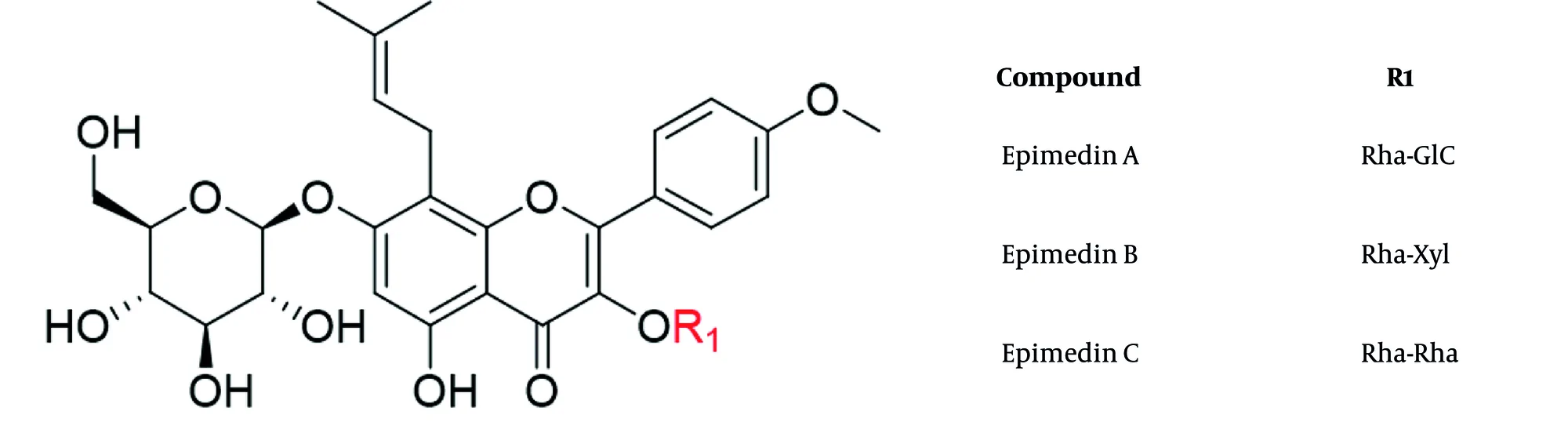
Chemical structures of epimedin A, epimedin B, and epimedin C. Abbreviations: glc, glucose; rha, rhamnose; xyl, xylose.

Herba Epimedii, commonly known as Yin Yang Huo, is a traditional Chinese medicinal herb used to treat various diseases (1). Phytochemical studies have shown that flavonoids (secondary plant metabolites that exist as glycosides), such as EA, EB, and EC, are its main active constituents (2, 3). These flavonoids share a common structural skeleton but differ in the glycosidic substituents attached at the C-3 position, as illustrated in Figure 1 (2). EA contains rhamnose and glucose, EB carries rhamnose and xylose linked by an α-1,2-glycosidic bond, and EC possesses two rhamnose units linked in a similar manner. Such structural variations may influence their physicochemical properties, protein-binding behavior, and ultimately their pharmacokinetic profiles (4). These flavonoids show considerable potential for the treatment of conditions such as osteoporosis, cardiovascular disease, sexual dysfunction, neurodegenerative diseases, and cancer (5-9). EB directly binds and stabilizes estrogen receptor ESR1, downregulates MAPK and PI3K-AKT phosphorylation, activates the AMPK-Nrf2 axis to reduce reactive oxygen species levels, and suppresses RANKL-induced osteoclast differentiation, thereby reversing bone loss in ovariectomized mice (10). EA inhibits osteoclastogenesis by negatively regulating TRAF6 expression and suppressing the PI3K/AKT/NF-κB axis (11). Beyond bone metabolism, these flavonoids also show cardiovascular, neuroprotective, and immunomodulatory potential (12). Regarding target engagement, EB and EC bind to the transmembrane domain of the calcium-sensing receptor, whereas EA and icariin, a related flavonoid glycoside also derived from Epimedium species, interact with its extracellular domain, indicating that subtle structural differences lead to distinct binding modes (13). From a structure-activity relationship perspective, glycosylation at the C-3 and C-7 positions is a key determinant of target selectivity and activity. The 7-O-glucose moiety reduces oral bioavailability, whereas its removal yields more potent metabolites (14). Furthermore, EC and icariin exhibit osteogenic effects by elevating alkaline phosphatase activity and reducing the RANKL/OPG ratio, whereas EA lacks such activity, suggesting that sugar composition critically modulates osteogenic potential (15). These findings demonstrate that, despite sharing a common aglycone backbone, distinct glycosylation patterns confer significant differences in target binding, signaling modulation, and therapeutic efficacy. Owing to their high efficacy and low toxicity, flavonoids represent promising drug candidates (16). To assess their clinical potential, it is essential to understand their pharmacokinetic profiles, including bioavailability and systemic distribution.

Human serum albumin, the most abundant plasma protein, is a key determinant of drug pharmacokinetics and serves as a central carrier that modulates drug distribution, metabolism, and efficacy (17-20). Structurally, HSA is a 66.5 kDa single-chain protein stabilized by 17 disulfide bonds and comprises three homologous α-helical domains (I, II, and III), each divided into two subdomains (A and B) (21, 22). The principal drug-binding sites are located in subdomains IIA (Sudlow site I) and IIIA (Sudlow site II) (23). Within subdomain IIA, the binding cavity is predominantly hydrophobic, whereas positively charged side chains, including Lys195, Arg218, and Arg222, are situated near the pocket entrance (22). This arrangement, featuring a polar rim and a hydrophobic core, may facilitate the accommodation of flavonoids with both aromatic ring systems and hydrophilic substituents. In contrast, the pocket within subdomain IIIA is hydrophobic (24) and may be suitable for flavonoids with relatively condensed molecular frameworks. Beyond its static architecture, HSA exhibits pH-dependent conformational flexibility known as the N-B transition (25), and its binding properties can be further modulated by allosteric effects induced by fatty acid occupation at neighboring sites (21, 23). Despite the pharmacological importance of epimedin flavonoids and the central role of HSA in pharmacokinetics, their interactions have not been systematically investigated.

UV-vis spectrophotometry is commonly used to study drug-protein interactions. Qualitative or quantitative analysis of absorption spectra resulting from drug binding to proteins can provide insights into protein-drug interactions (26). *Fluorescence* spectroscopy is another technique used to probe drug-protein interactions. Drug binding may alter the fluorescence and synchronous fluorescence spectra of a protein, thereby helping to elucidate the interaction mechanism, quenching mechanism, and binding affinity (27, 28). Synchronous fluorescence measurements are also useful for assessing the polarity of the microenvironment around aromatic amino acids and investigating conformational changes in proteins (29, 30).

## 2. Objectives

To address this knowledge gap, this study investigated the interactions of EA, EB, and EC with HSA under simulated physiological conditions using UV-vis spectrophotometry, fluorescence spectroscopy, synchronous fluorescence spectroscopy, molecular docking, and molecular dynamics simulations. The findings were intended to provide preliminary in vitro insights into the transport and distribution profiles of these flavonoids and to establish a basis for future studies of their pharmacokinetic and disposition characteristics under more physiologically relevant conditions.

## 3. Methods

### 3.1. Reagents

EA (purity > 99%), EB (purity > 99%), and EC (purity > 99%) were purchased from the Chinese National Institute for the Control of Pharmaceutical and Biological Products (Beijing, China). HSA was purchased from Sigma-Aldrich Chemical Company (St. Louis, MO, USA). All chemicals used in this study were of analytical reagent grade.

### 3.2. Preparation of Stock Solutions

An HSA stock solution (1.0 × 10^-5^ mol·L^-1^) was prepared by dissolving 0.0668 g of HSA in 100 mL of distilled water and storing it at 4 °C in the dark. Tris-HCl buffer (pH 7.4, 0.05 mol·L^-1^) was prepared. A 0.5 mol·L^-1^ solution was prepared by dissolved 1.4602 g of NaCl in distilled water and diluting to a final volume of 50 mL. Stock solutions of EA, EB, and EC (approximately 2.0 × 10^-3^ mol·L^-1^) were prepared in methanol. Standard working solutions were prepared by further dilution of the stock solution to the required concentrations. All solutions were freshly prepared. 

### 3.3. UV-Vis Spectroscopy

UV-vis absorption spectra were recorded using a UV-2450 spectrophotometer (Shimadzu, Japan) with 1.0 cm quartz cells. The stock solutions were diluted with distilled water to the desired concentrations. HSA was mixed with EA, EB, or EC and transferred to the quartz cells. UV-vis absorption spectra were scanned over the wavelength range of 250 - 360 nm at 298 K.

### 3.4. *Fluorescence* Spectroscopy

*Fluorescence* spectroscopy was performed using an RF-540 fluorescence spectrophotometer (Shimadzu, Japan) with 1.0 cm quartz cells. The HSA stock solution was mixed with Tris-HCl buffer (pH 7.4) and diluted with distilled water. The mixtures were added to quartz cuvettes, followed by the addition of EA, EB, or EC stock solution, and allowed to react for 5 min. *Fluorescence emission* spectra were recorded at 298, 303, and 310 K over the range of 300 - 450 nm at an excitation wavelength of 278 nm, with excitation and emission slit widths of 5 nm.

### 3.5. Synchronous *Fluorescence* Spectroscopy

The instrument described in section 3.4 was used to obtain synchronous fluorescence spectra. The HSA concentration was kept constant, whereas the concentrations of the three flavonoids were gradually increased. The protein-flavonoid mixtures were allowed to react for 5 min before spectral acquisition. Synchronous fluorescence spectra were recorded at Δλ = 60 nm and Δλ = 15 nm. Here, Δλ is the difference between the emission and excitation wavelengths, i.e., Δλ = λ_em_ - λ_ex_. The excitation and emission slit widths were 5 nm.

### 3.6. Molecular Docking

Ligand preparation: The structures of the active ingredients were obtained from the PubChem and ChemSpider databases. Energy minimization and geometric optimization were performed using ChemBio3D software. Receptor preparation: The HSA structure was downloaded from the RCSB PDB database (PDB ID: 1BKE, http://www.rcsb.org/) and processed using AutoDockTools (ADT) 1.5.6 for hydrogenation, Gasteiger charge assignment, and atomic-type definition. Docking analysis: The protein active site was defined in ADT, and molecular docking was performed using AutoDock Vina.

### 3.7. Molecular Dynamics Simulations

Molecular dynamics simulations of the nine HSA-flavonoid complexes were performed using GROMACS 2022.2. HSA was parameterized with the Amber99SB-ILDN force field, whereas ligand parameters were generated using Sobtop 1.0 (dev3.1) with the GAFF2 force field and RESP atomic charges. Each complex was solvated in a cubic TIP3P water box with a minimum protein-to-box distance of 1.0 nm, neutralized with Na^+^ and Cl^-^, and adjusted to 0.15 M NaCl. Long-range electrostatic interactions were treated using the particle mesh Ewald method, with cutoff distances of 1.2 nm for short-range electrostatic and van der Waals interactions. Covalent bonds were constrained using LINCS, and a 2 fs time step was applied. After steepest-descent and conjugate-gradient energy minimization, each system underwent 1 ns NVT and 1 ns NPT equilibration at 300 K and 1 bar, followed by an unrestrained 100 ns production simulation. Root-mean-square deviation (RMSD), root-mean-square fluctuation (RMSF), radius of gyration, solvent-accessible surface area, and free-energy landscapes were analyzed using GROMACS tools. Binding free energies and residue contributions were evaluated using the MM/PBSA method.

## 4. Results

### 4.1. UV-Vis Spectrophotometry

**Figure 2.**
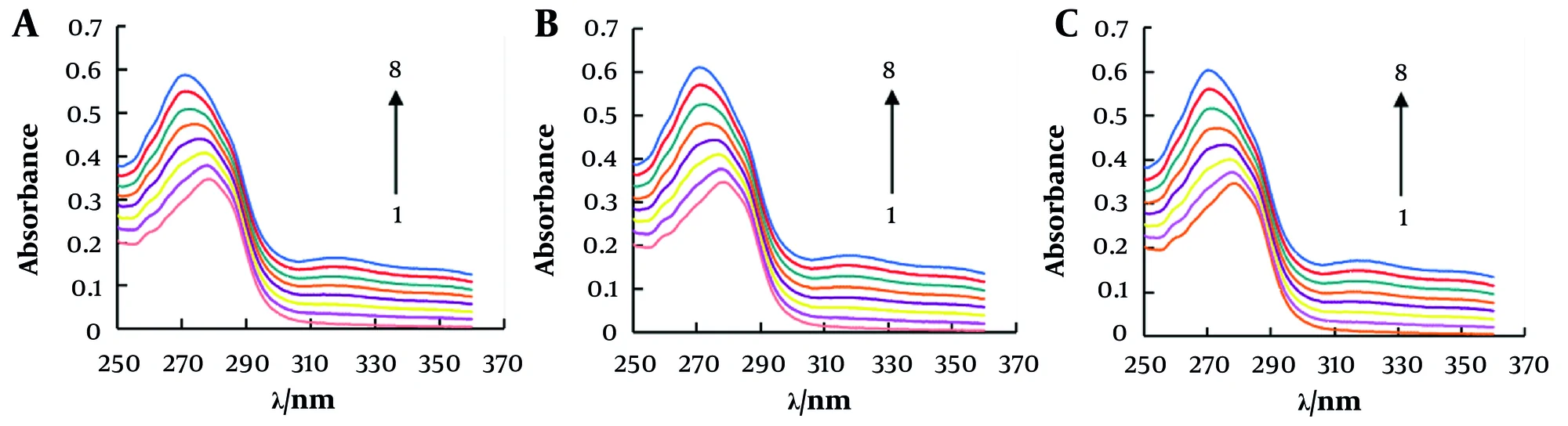
UV-absorption spectra of the interaction of EA (A), EB (B), and EC (C) with HSA. C_HSA_ = 10^-5^ mol·L^-1^; C_NaCl_ = 0.5 mol·L^-1^. C_EA_ = 0, 2 × 10^-6^, 4 × 10^-6^, 6 × 10^-6^, 8 × 10^-6^, 1 × 10^-5^, 1.2 × 10^-5^, 1.4 × 10^-5^ mol·L^-1^; C_EB_ = 0, 2 × 10^-6^, 4 × 10^-6^, 6 × 10^-6^, 8 × 10^-6^, 1 × 10^-5^, 1.2 × 10^-5^, 1.4 × 10^-5^; C_EC_ = 0, 2.07 × 10^-6^, 4.13 × 10^-6^, 6.20 × 10^-6^, 8.26 × 10^-6^, 1.03 × 10^-5^, 1.24 × 10^-5^, 1.44 × 10^-5^ mol·L^-1^.

HSA absorption is derived from tryptophan (Trp), tyrosine (Tyr), and phenylalanine (Phe). HSA has a characteristic absorption peak near 280 nm resulting from the π-π* transitions of Trp, Tyr, and Phe residues (31-33). The UV absorption spectra of HSA in the presence of EA, EB, and EC are shown in Figure 2A-C. Upon addition of all three flavonoids, this band showed a consistent hyperchromic effect (increased intensity) accompanied by a slight but discernible blue shift. These spectral alterations indicate ligand–protein complex formation. Specifically, the blue shift suggests that the microenvironment surrounding the aromatic chromophores of HSA becomes less polar upon flavonoid binding, possibly because of insertion into hydrophobic protein pockets or a subtle conformational change that reduces solvent exposure of these residues. The spectral changes induced by EA, EB, and EC were qualitatively similar, suggesting analogous initial interaction modes with the protein scaffold.

### 4.2. Quenching of HSA *Fluorescence* in the Presence of EA, EB, and EC

**Figure 3.**
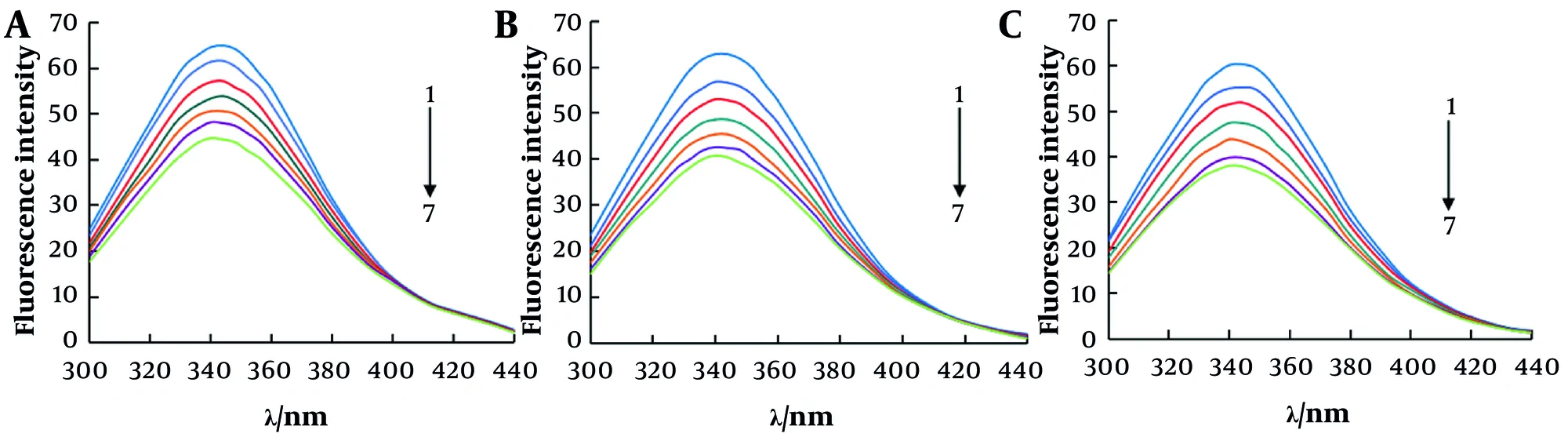
*Fluorescence emission* spectra of HSA with different concentrations of three flavonoids at 298 K. (A) EA, (B) EB, (C) EC. C_EA_ = 0, 2 × 10^-6^, 4 × 10^-6^, 6 × 10^-6^, 8 × 10^-6^, 1 × 10^-5^, 1.2 × 10^-5^ mol·L^-1^; C_EB_ = 0, 2 × 10^-6^, 4 × 10^-6^, 6 × 10^-6^, 8 × 10^-6^, 1 × 10^-5^, 1.2 × 10^-5^ mol·L-1; C_EC_ = 0, 2.07 × 10^-6^, 4.13 × 10^-6^, 6.20 × 10^-6^, 8.26 × 10^-6^, 1.03 × 10^-5^, 1.24 × 10^-5^ mol·L-1.

Trp, Tyr, and Phe residues emit endogenous fluorescence, with fluorescence maxima at 348, 303, and 282 nm, respectively (34). Because the fluorescence of Phe and Tyr is weak, the fluorescence spectrum of HSA is primarily dominated by Trp residues located in subdomain IIA. The fluorescence spectra of HSA at 298 K in the presence of EA, EB, and EC, upon excitation at 280 nm, are shown in Figure 3A-C. In the absence of any flavonoid, HSA exhibited a fluorescence maximum at approximately 344 nm (35). The fluorescence maxima were slightly blue-shifted after the addition of EA, EB, and EC and were accompanied by significant quenching of fluorescence intensity as the concentrations of these flavonoids increased. These findings are consistent with an association between EA, EB, or EC and HSA under the tested conditions. The slight blue shift may reflect a subtle change in polarity or solvent accessibility of the environment surrounding Trp214, potentially arising from local or long-range conformational effects (36, 37).

### 4.3. *Fluorescence* Quenching Mechanism

*Fluorescence* quenching can result from molecular interactions, including excited-state reactions, ground-state complex formation, energy transfer, and collisional quenching (38). *Fluorescence* can be quenched through static or dynamic mechanisms, or a combination of both (39). Dynamic quenching results from collisions between the quencher and excited-state fluorophores, leading to deactivation of the fluorophore to the ground state. It affects only the excited state of the molecules and does not change the absorption spectrum of the fluorophore (40). In static quenching, the quencher and fluorophore form a nonfluorescent ground-state complex before the fluorophore is excited (41). The two mechanisms can be distinguished based on quenching behavior at different temperatures. In dynamic quenching, increasing temperature increases the diffusion coefficient, particle motion, and energy transfer; therefore, the quenching constant increases with temperature. In static quenching, the quenching constant decreases because the stability of the nonfluorescent complex decreases with increasing temperature (31, 41, 42).

The quenching mechanism of the HSA complexes can be determined from fluorescence intensities at different temperatures (298, 303, and 310 K) using the Stern-Volmer equation (Equation 1) (43, 44).

Equation 1.

$$\frac{F_{0}}{F}=1+K_{q}\tau_{0}\left[Q\right]=1+K_{\mathrm{SV}}\mathrm{[Q]}$$

**Figure 4.**
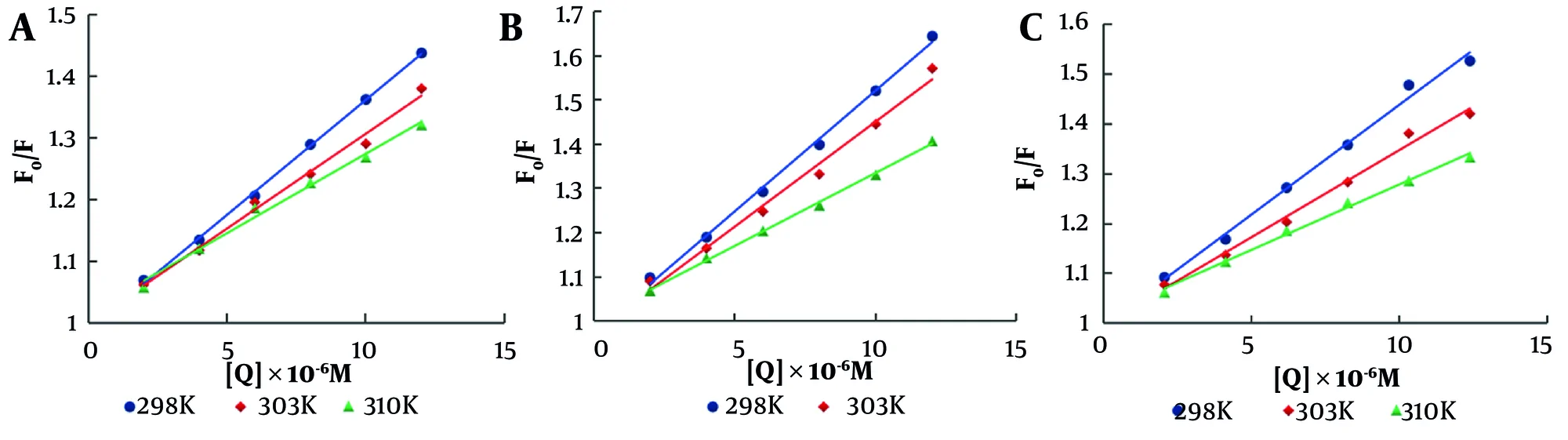
Stern-Volmer plots for determining quenching constants of HSA in the presence of different concentrations of EA (A), EB (B), and EC (C) at 298, 303, and 310 K.

Here, F_0_ represents the fluorescence intensity of the protein, F is the fluorescence intensity of the protein in the presence of a drug, K_q_ is the bimolecular quenching constant, K_SV_ is the Stern-Volmer quenching constant, [Q] is the concentration of EA, EB, or EC, and τ_0_ is the fluorescence lifetime of the fluorophore (approximately 10^-8^ s). The Stern-Volmer curves of the EA-HSA, EB-HSA, and EC-HSA complexes are shown in Figure 4A-C. For all three flavonoids, the slopes of the linear plots decreased with increasing temperature, indicating a static quenching mechanism. The corresponding linear equations and constants are listed in Table 1. The maximum diffusion collision quenching rate constant between different quenchers and biological macromolecules is 2.0 × 10^10^ L·mol^-1^·s^-1^ (45, 46). However, the fluorescence quenching constants in the present study were much larger than this value, further supporting a static rather than dynamic quenching mechanism.

**Table 1.** Stern-Volmer Quenching Constants of HSA Complexes at Different Temperatures

Compounds and T (K)	Stern-Volmer equation (y)	K_SV_ (× 10^4^ L/mol)	K_q_ (× 10^12^ L/mol/s)	R^2^
**EA**				
298	0.0373x + 0.9901	3.73	3.73	0.9983
303	0.0308x + 0.999	3.08	3.08	0.9914
310	0.0258x + 1.0167	2.58	2.58	0.9911
**EB**				
298	0.0546x + 0.9741	5.46	5.46	0.9968
303	0.0474x + 0.9782	4.74	4.74	0.9933
310	0.033x + 1.0043	3.30	3.30	0.9979
**EC**				
298	0.044x + 0.9975	4.40	4.40	0.9926
303	0.0349x + 0.9977	3.49	3.49	0.9910
310	0.0262x + 1.0159	2.62	2.62	0.9949

### 4.4. Thermodynamic Analysis of Binding Affinity

For a static quenching process, the binding constant K and the number of binding sites n between a drug and a protein can be determined using the following double-logarithmic equation (Equation 2):

Equation 2.

$$\log\left(\frac{F_{0}-F}{F}\right)=\mathrm{LogK}+\mathrm{nlog[Q]}$$

**Figure 5.**
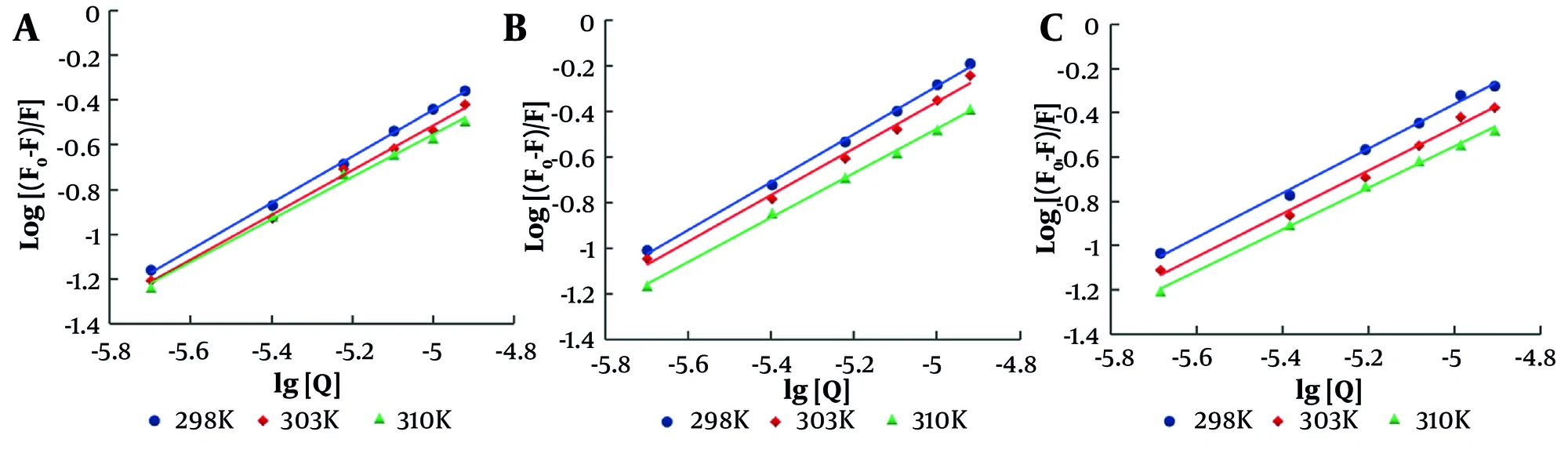
Double-logarithm plots of the EA-HSA system (A), EB-HSA system (B), and EC-HSA system (C) at 298, 303, and 310 K.

F_0_, F, and [Q] have the same meanings as in Equation (1). By plotting the corresponding double-logarithmic relationship, the slope (n) and intercept can be obtained at 298, 303, and 310 K, as shown in Figure 5A-C. As shown in Table 2, the binding constants K for the HSA-flavonoid systems at the three temperatures all exceeded 10^4^, indicating moderate binding affinity between HSA and EA, EB, and EC (47). The binding-affinity data indicate that these three flavonoids can form stable complexes with HSA, suggesting that they could be transported via HSA in the circulatory system (47). Moreover, K decreased with increasing temperature, confirming a static quenching mechanism. The number of binding sites (n) on HSA for EA, EB, and EC was approximately 1, indicating that each flavonoid formed a 1:1 complex with HSA. At 298 K, binding affinity followed the order EB > EA > EC. This hierarchy suggests that the specific glycosyl composition at the C-3 position influences binding strength. The relatively stronger binding of EB might be tentatively attributed to the structural properties of its xylose moiety compared with the glucose in EA or the second rhamnose in EC, a hypothesis to be explored further through molecular docking.

**Table 2.** Binding Constants of HSA Complexes at Different Temperatures

Compounds and T (K)	Linear equation (y)	K (× 10^4^ L/mol)	n	R^2^
**EA**				
298	1.041x + 4.7634	5.80	1.041	0.9987
303	0.9984x + 4.4772	3.00	0.9984	0.9956
310	0.9496x + 4.1939	1.56	0.9496	0.9938
**EB**				
298	1.052x + 4.9701	9.33	1.052	0.9980
303	1.0241x + 4.7662	5.84	1.0241	0.9945
310	0.9746x + 4.3974	2.50	0.9746	0.9984
**EC**				
298	1.0043x + 4.6616	4.59	1.0043	0.9958
303	0.9751x + 4.4108	2.58	0.9751	0.9931
310	0.941x + 4.1546	1.43	0.941	0.9979

### 4.5. Thermodynamic Parameters of Binding Between Flavonoids and HSA

Drug-protein interactions mainly include hydrogen bonding, van der Waals forces, electrostatic interactions, and hydrophobic interactions. The thermodynamic constants for the interactions between HSA and EA, EB, and EC can be calculated from the binding constants at different temperatures using the Van't Hoff equation (48):

Equation 3.

$$\mathrm{lnK}=\frac{-∆H^{0}}{\mathrm{RT}}+\frac{∆S^{0}}{R}$$

Equation 4.

$$∆G^{0}=∆H^{0}-\mathrm{T∆}S^{0}$$

ΔH^0^, ΔS^0^, and ΔG^0^ are the enthalpy change, entropy change, and standard molar Gibbs free energy change of the reaction, respectively. R is the gas constant (8.314 J·K^-1^·mol^-1^), and T is the absolute temperature. By plotting lnK against 1/T, the slope and intercept can be obtained. Subsequently, ΔG^0^ can be calculated according to Equation (4). As reported previously, if ΔH^0^ > 0 and ΔS^0^ > 0, binding is dominated by hydrophobic forces; if ΔH^0^ < 0 and ΔS^0^ > 0, electrostatic forces dominate; and if ΔH^0^ < 0 and ΔS^0^ < 0, binding is dominated by hydrogen bonding and van der Waals forces (39).

Table 3 lists the thermodynamic parameters for the binding of EA, EB, and EC to HSA. For all three flavonoids, ΔH^0^ < 0, ΔS^0^ < 0, and ΔG^0^ < 0 at 298, 303, and 310 K, indicating a spontaneous, enthalpy-driven process governed by hydrogen bonding and van der Waals interactions. The relatively large negative ΔS^0^ values may reflect a substantial loss of translational, rotational, and conformational freedom upon complex formation. In particular, binding may restrict the flexible glycosidic moieties of EA, EB, and EC and promote ordering of interfacial solvent molecules or hydrogen-bond networks.

**Table 3.** Thermodynamic Parameters for HSA-Flavonoid Systems at Different Temperatures

Compounds and T (K)	ΔG^0^ (kJ/mol)	ΔH^0^ (kJ/mol)	ΔS^0^ (J/K/mol)
**EA**		-83.3063	-188.6779
298	-27.0803		
303	-26.1369		
310	-24.8161		
**EB**		-84.9774	-189.7255
298	-28.4392		
303	-27.4906		
310	-26.1625		
**EC**		-74.2207	-160.0694
298	-26.5200		
303	-25.7197		
310	-24.5992		

### 4.6. Synchronous *Fluorescence* Measurements

**Figure 6.**
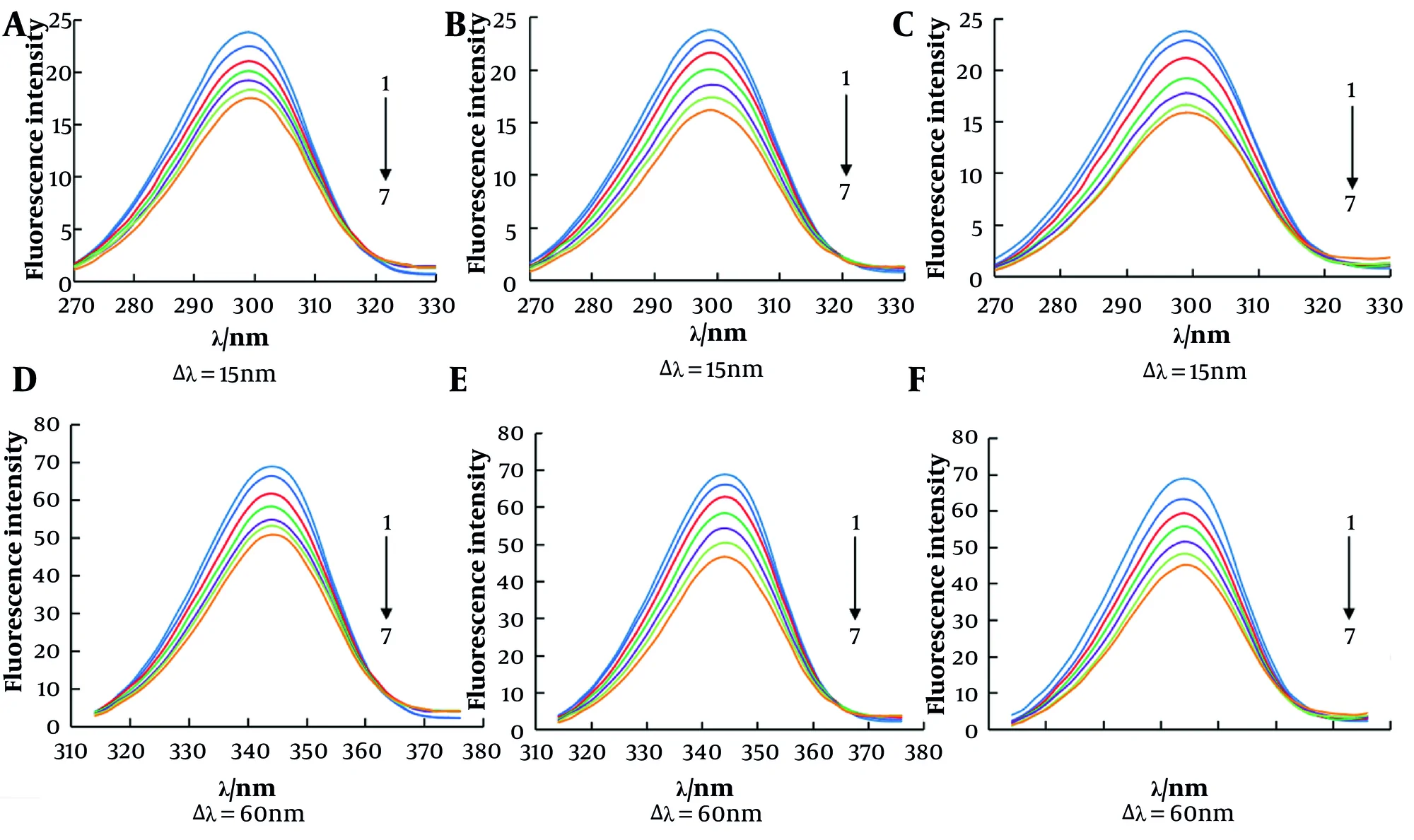
Synchronous fluorescence spectra of HSA in the presence of different concentrations of EA (A, D), EB (B, E), and EC (C, F). C_EA_ = 0, 2 × 10^-6^, 4 × 10^-6^, 6 × 10^-6^, 8 × 10^-6^, 1 × 10^-5^, 1.2 × 10^-5^ mol·L^-1^; C_EB_ = 0, 2 × 10^-6^, 4 × 10^-6^, 6 × 10^-6^, 8 × 10^-6^, 1 × 10^-5^, 1.2 × 10^-5^ mol·L^-1^; C_EC_ = 0, 2.07 × 10^-6^, 4.13 × 10^-6^, 6.20 × 10^-6^, 8.26 × 10^-6^, 1.03 × 10^-5^, 1.24 × 10^-5^ mol·L^-1^.

Synchronous fluorescence spectra of HSA at various concentrations of EA, EB, and EC, with Δλ = 15 nm and Δλ = 60 nm, are shown in Figure 6A-F. When Δλ = 15 and 60 nm, synchronous fluorescence is characteristic of Tyr and Trp residues, respectively. HSA fluorescence intensity decreased significantly after the addition of EA, EB, and EC, with no peak shift at Δλ = 15 nm or Δλ = 60 nm. Thus, the addition of the flavonoids did not induce major changes in the Tyr and Trp microenvironments. This observation, combined with the blue shifts observed in UV-vis and steady-state fluorescence, suggests that binding may induce subtle conformational changes affecting the electronic state of the chromophores without altering the local hydrophobicity around specific aromatic side chains. These findings are compatible with subtle long-range conformational or allosteric effects, but the present spectroscopic data cannot distinguish these effects from proximity-based quenching.

### 4.7. Molecular Docking

**Figure 7.**
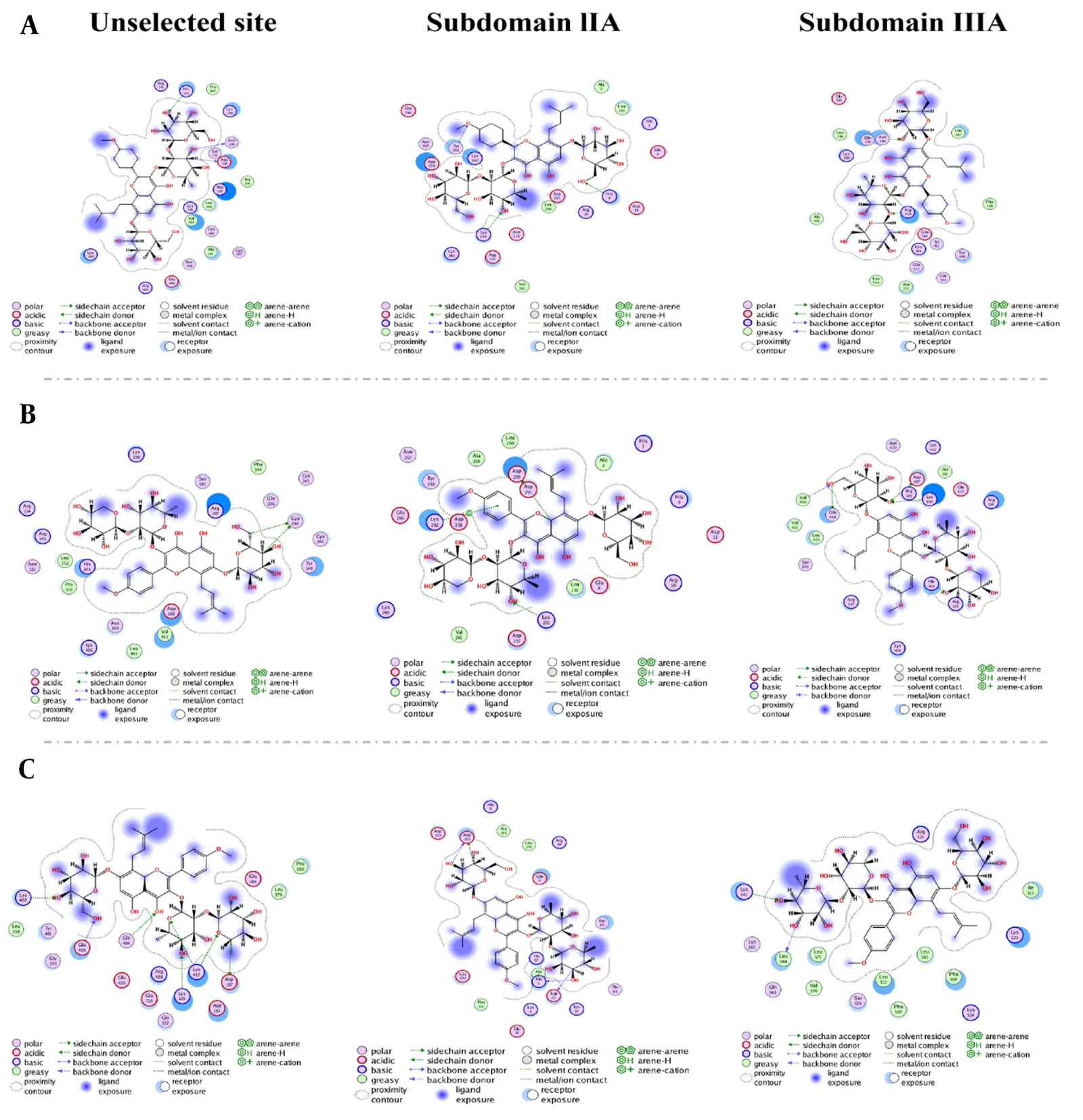
Molecular modeling of the interaction between EA (A), EB (B), and EC (C) with HSA.

Molecular docking studies were performed to determine the major binding sites of these flavonoids and to better understand their interactions with HSA (49, 50). To elucidate the binding patterns and interactions of EA, EB, and EC with the target protein, molecular docking assays were performed against two well-characterized binding sites (IIA and IIIA) reported in the literature, together with an unselected-site docking strategy for comparison. As shown in Figure 7A-C, the 2D ligand-residue interaction diagrams revealed divergent interaction networks formed by EA, EB, and EC within the nonclassical binding pocket. EA formed hydrogen bonds with His146, Asn109, and Asp108 and engaged in extensive van der Waals contacts with surrounding polar, basic, and hydrophobic amino acid residues. EB formed side-chain hydrogen bonds with Cys200, with a dense cluster of polar and aliphatic residues surrounding its molecular skeleton. EC established an extensive hydrogen-bond network through interactions with Lys402, Glu400, Gln404, Lys432, Lys519, and Asp187. By contrast, only sparse hydrogen bonds were observed at Sudlow sites IIA and IIIA. The docking results showed that all three compounds had their most favorable binding affinities at the unselected site, with binding energies of -9.302, -8.448, and -8.395 kcal/mol for EA, EB, and EC, respectively. Their binding affinities decreased when docked to the specific IIA and IIIA sites: binding energies at site IIA were -8.129, -7.704, and -7.617 kcal/mol, whereas those at site IIIA were -7.766, -7.285, and -6.801 kcal/mol for EA, EB, and EC, respectively. These findings suggest that the IIA and IIIA sites might represent narrow active pockets that cannot accommodate the three compounds well, resulting in weaker binding interactions than those observed with the unselected-site docking mode.

### 4.8. Molecular Dynamics Simulation Analysis

**Figure 8.**
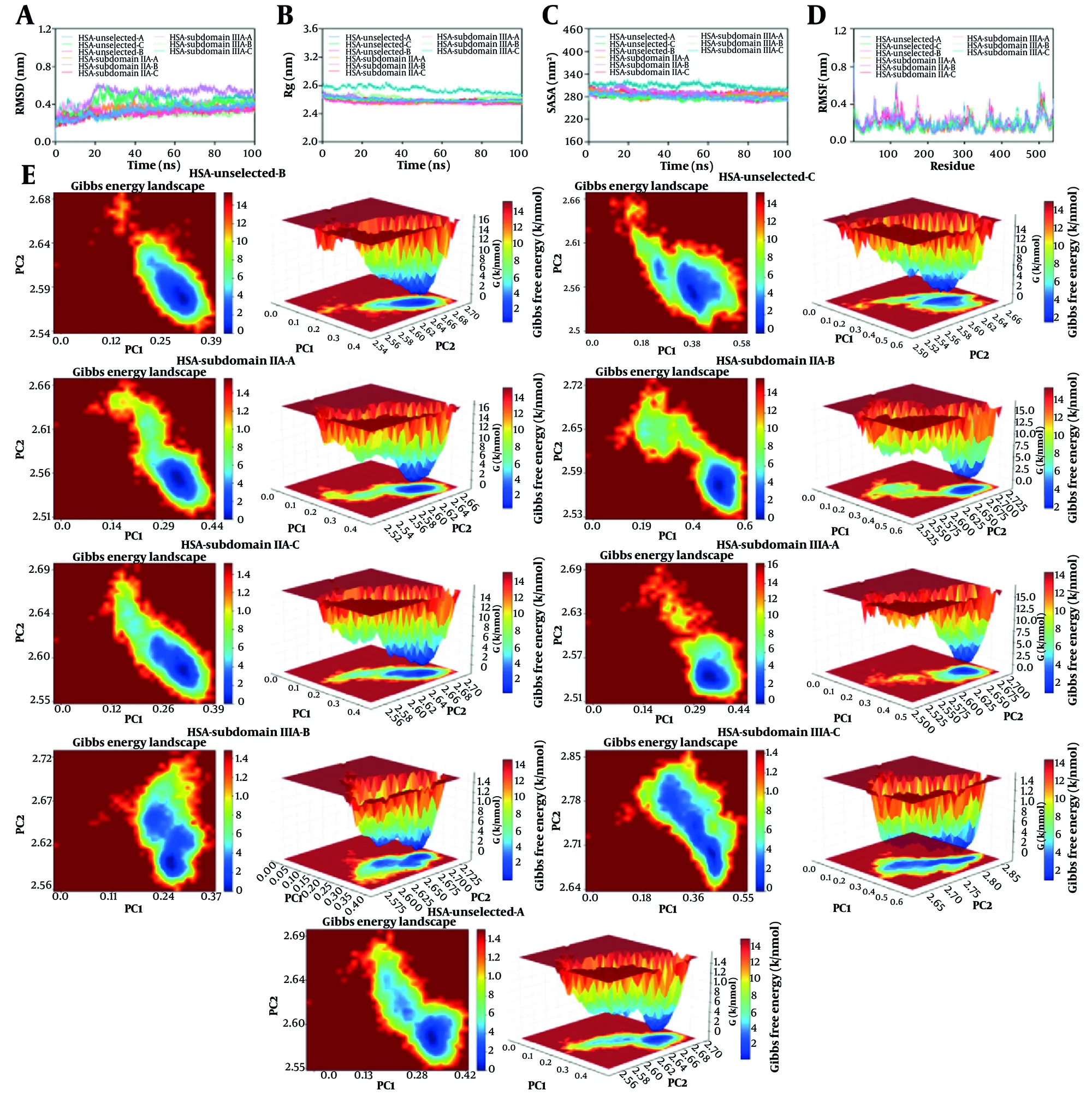
Molecular dynamics simulations of the protein-ligand complexes. (A) Time-dependent RMSD of the protein-ligand complexes; (B) time-dependent radius of gyration (Rg); (C) time-dependent solvent-accessible surface area (SASA); (D) RMSF of the protein-ligand complexes; and (E) free-energy landscape (FEL) analysis.

The dynamic stability of the nine HSA-flavonoid complexes was evaluated using 100 ns molecular dynamics simulations, as shown in Figure 8A-E. The RMSD trajectories of all systems gradually reached relatively stable plateaus. For the whole-protein blind-docking complexes, the EA, EB, and EC systems reached equilibrium after approximately 70, 80, and 25 ns, respectively, with final RMSD values fluctuating around 0.42, 0.40, and 0.42 nm. The corresponding equilibrium RMSD values for the subdomain IIA complexes were approximately 0.41, 0.57, and 0.35 nm, whereas those for the subdomain IIIA complexes were approximately 0.37, 0.36, and 0.50 nm, respectively. The radius-of-gyration profiles remained relatively stable throughout the simulations, indicating that ligand binding did not induce marked expansion or contraction of the overall HSA structure. Similarly, no substantial changes were observed in the solvent-accessible surface area of the complexes. Most RMSF values were below approximately 0.65 nm, suggesting limited residue-level fluctuations and generally stable protein conformations. Free-energy landscape analysis identified distinct low-energy basins for all nine complexes, indicating dominant stable conformational states during the simulations. MM/PBSA analysis yielded binding free energies of -25.01, -31.52, and -10.81 kcal/mol for the whole-protein EA, EB, and EC complexes, respectively. The corresponding values for the subdomain IIA complexes were -26.33, -9.51, and -30.19 kcal/mol, whereas those for the subdomain IIIA complexes were -12.03, -35.75, and -26.16 kcal/mol, respectively. All calculated binding free energies were negative, supporting an energetically favorable association between the flavonoids and HSA. However, the energetically preferred candidate binding region differed among EA, EB, and EC. Residue-energy decomposition further identified amino acid residues that contributed substantially to ligand binding. In the whole-protein blind-docking complexes, major contributing residues included Lys466, Arg197, Leu463, Val462, Gln459, Ser193, and Asn109 for EA; Arg197, Tyr148, Ser193, and Gln196 for EB; and Pro180 for EC. Different residue-contribution patterns were observed for the subdomain IIA and IIIA complexes, suggesting that the three flavonoids were stabilized by distinct local residue networks.

## 5. Discussion

The present findings provide preliminary in vitro evidence regarding the binding behavior of epimedin flavonoids to HSA. However, in vitro experiments with HSA cannot reproduce the complexity of the plasma environment. Endogenous ligands, concomitant drugs, plasma proteins, ionic conditions, pH variations, and structural modifications of HSA may alter albumin binding in vivo.

### 5.1. Study Limitations and Future Directions

Albumin binding alone does not determine absorption, tissue distribution, clearance, or pharmacological properties. Further studies in plasma matrices, together with competitive-binding, pharmacokinetic, and in vivo investigations, are required to determine the physiological and translational relevance of these interactions. Future work should also examine the influence of more complex physiological factors, such as variable pH, the presence of metal ions or competing ligands, and their effects on HSA structure or esterase-like activity.

### 5.2. Conclusions

EA, EB, and EC quenched the endogenous fluorescence of HSA via a static quenching mechanism. The binding constants between the three flavonoids and HSA were all greater than 10^4^, indicating moderate binding to HSA. HSA had one binding site for EA, EB, and EC. Binding was spontaneous and mainly dominated by hydrogen bonding and van der Waals interactions. The microenvironment of the Trp residues in HSA was not significantly affected by flavonoid binding.

## Data Availability

The dataset presented in the study is available on request from the corresponding author during submission or after publication.
